# First case report of a vertebral osteomyelitis caused by carbapenem-resistant *Enterobacter cloacae* treated with imipenem/cilastatin/relebactam prolonged infusion then meropenem/vaborbactam in continuous infusion

**DOI:** 10.3389/fphar.2024.1347306

**Published:** 2024-10-30

**Authors:** Paul Laffont-Lozes, Tayma Naciri, Alix Pantel, Aurélie Martin, Anne-Sophie Pruvot-Occean, Vincent Haignere, Paul Loubet, Albert Sotto, Romaric Larcher

**Affiliations:** ^1^ Department of Pharmacy, Nimes University Hospital, Nîmes, France; ^2^ Department of Infectious and Tropical Diseases, Nimes University Hospital, Nîmes, France; ^3^ Department of Microbiology and Hospital Hygiene, Nimes University Hospital, Nîmes France; ^4^ VBIC (Bacterial Virulence and Chronic Infection), INSERM (French Institute of Health and Medical Research), Montpellier University, Montpellier, France; ^5^ Department of Neurosurgery, Nimes University Hospital, Nîmes, France; ^6^ Department of Orthopaedic Surgery and Traumatology, Nimes University Hospital, Nîmes, France; ^7^ PhyMedExp (Physiology and Experimental Medicine), INSERM (French Institute of Health and Medical Research), CNRS (French National Centre for Scientific Research), University of Montpellier, Montpellier, France

**Keywords:** bone and joint infection, vertebral osteomyelitis, meropenem/vaborbactam, continuous infusion, imipenem/cilastatin/relebactam, extended infusion, therapeutic drug monitoring

## Abstract

**Introduction:**

Bone and joint infections (BJIs) caused by multidrug-resistant bacteria are becoming more frequent. However, data on the use of novel β-lactam/β-lactamase inhibitors, such as imipenem/cilastatin/relebactam (I-R) and meropenem/vaborbactam (MVB), to treat BJIs is lacking. Furthermore, prolonged infusions of these β-lactams should theoretically optimize pharmacokinetic/pharmacodynamics target in these indications, but there are currently no reports on this type of infusions, especially in the setting of BJI.

**Case Presentation:**

We report a case of a vertebral osteomyelitis caused by carbapenem-resistant *Enterobacter cloacae* successfully treated with extended-infusion of I-R (1.25 g q6h over 2 h), then with continuous infusion of MVB (2 g q4h as over 4 h). Therapeutic drug monitoring confirmed that extended-infusion of I-R and continuous infusion of MVB achieved serum concentrations up to 12 mg/L of imipenem and 19 mg/L of meropenem, respectively.

**Conclusion:**

The favourable outcome of this patient treated for a vertebral osteomyelitis caused by carbapenem-resistant *E. cloacae* suggest that extended- and continuous infusions of I-R and MVB, are promising regimens for treatment of BJIs caused by carbapenem-resistant Enterobacterales.

## 1 Introduction

The incidence of multidrug resistance (MDR) is increasing worldwide, leading to higher mortality and longer hospital stays (Murray et al., 2022). In response to this issue, new antimicrobials have recently been developed. Novel β-lactam/β-lactamase inhibitors are now the first treatment option for carbapenem-resistant Gram-negative bacteria (Tamma et al., 2022). Meropenem/vaborbactam (MVB) and imipenem/cilastatin/relebactam (I-R) are antimicrobials combining a carbapenem and a new β-lactamase inhibitor active against carbapenemase. They are approved by the European Medicines Agency and/or the US Food and Drug Administration (Summary of product characteristics: Vaborem, 2018; Summary of product characteristics: Recarbrio, 2020), for the treatment of bacteraemia, hospital-acquired pneumonia including ventilator associated pneumonia, complicated urinary tract infection and complicated intra-abdominal infection. However, the use of these molecules in more complex infections such as bone and joint infections (BJIs) has not been studied.

Data on the use of these novel antimicrobials for treating BJIs are needed, as they represent a promising therapeutic option for managing BJIs caused by MDR bacteria, which are an increasing concern (Rebold et al., 2021; Rempenault et al., 2021; Larcher et al., 2022; Davido et al., 2023). In addition, previous data (Roberts et al., 2016; Rebold et al., 2021; Larcher et al., 2022; Le Vavasseur and Zeller, 2022) have suggested that it could be interesting to use these new β-lactams in prolonged or continuous infusion, to improve pharmacokinetic, bacteriological eradication and clinical success in BJIs caused by carbapenem-resistant *Enterobacterales*.

We aimed to report the first case of vertebral osteomyelitis caused by carbapenem-resistant *Enterobacter cloacae* successfully treated with extended infusion of I-R then continuous infusion of MVB. This case provides important data on using novel antimicrobials for BJIs and offers insights into their optimized administration for effective management.

## 2 Case description

**FIGURE 1:**
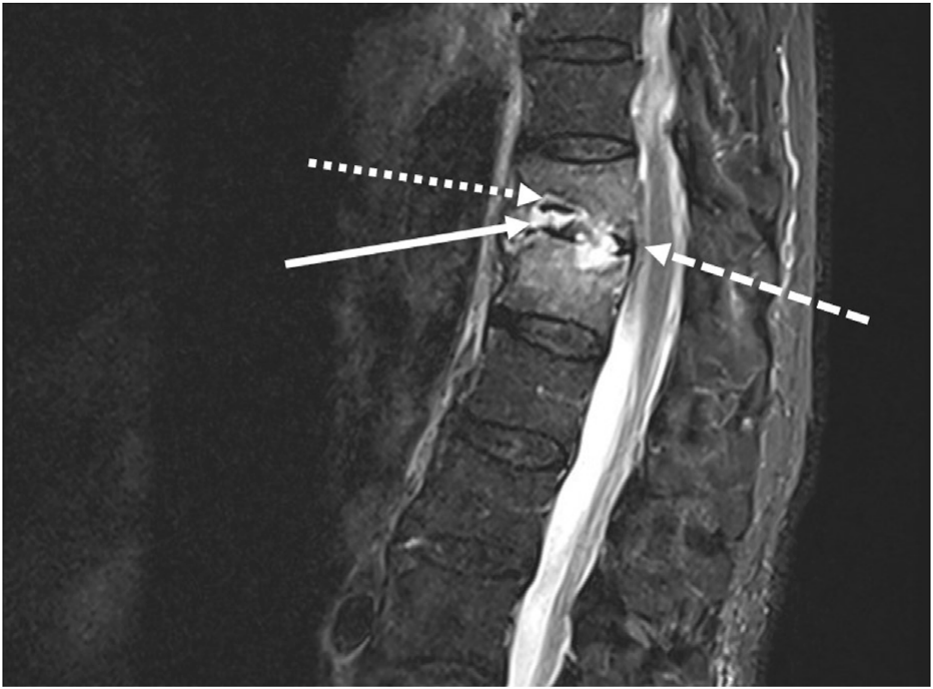
Medullar magnetic resonance imaging: Decreased height and STIR hypersignal of disc (white arrow) with irregularity and erosion of T11-T12 vertebral body endplates (white dotted arrow), and epidural collections (white dashed arrow).

In November 2022, a 77-year-old man, weighing 78 kg, was admitted to a French tertiary hospital for opiate-resistant back pain. He had a medical history of hypertension, Parkinson’s disease and atrial fibrillation. Two months prior, he had also been treated with ofloxacin for 21 days, for prostatitis caused by extended spectrum beta-lactamase (ESBL) producing-*E. cloacae*. On admission to the emergency room, a neutrophil count of 8.3 G/L (N = 1.5–7 G/L) and C reactive protein (CRP) level at 87.5 mg/L (N < 0.5 mg/L) prompted the physicians to perform a lumbar magnetic resonance imaging (MRI). A T11-T12 vertebral osteomyelitis with a significant infiltration of the surrounding soft tissues and an epiduritis was found (Figure 1). The patient was admitted to the infectious disease department and a couple of days later, ertapenem 1 g q12 h was started when blood cultures flagged positive for Gram-negative bacilli. An ESBL-producing-*E. cloacae* (Table 1) was identified. After 8 days of ertapenem therapy, the patient still had fever and CRP level remained at a plateau of 70 mg/L. Blood cultures drawn at this point were positive for carbapenem-resistant *E. cloacae* (Table 1). No carbapenemase enzyme was found (Xpert^®^ Carba-R test, Cepheid, CA, United States and β CARBA test^®^, Bio-Rad, CA,USA) and resistance to ertapenem was explained by a combination of impermeability with ESBL and AmpC-beta-lactamase. Extended-infusion meropenem 2 g q8h over 3 h was started according to the 2022 Infectious Diseases Society of America (IDSA) guidelines (Tamma et al., 2022) for infections caused by non-carbapenemase producing carbapenem-resistant *Enterobacterales* (non-CP-CRE) resistant to ertapenem, that remain susceptible to meropenem (Table 1). On the first day of treatment, a single dose of amikacin (30 mg/kg) was also administered. Blood cultures were negative after 5 days of treatment. However, back pain and fever persisted, the patient became disoriented, and CRP level rose to 99 mg/L. Clinical failure and drug neurotoxicity related to the use of a high dose of meropenem despite reduced renal clearance (estimated glomerular filtration rate, eGFR, at around 40 mL/min/1.73 m^2^ at this point) were suspected. Therefore, we decided to change the antibiotic therapy.

**TABLE 1:** Antibiotic susceptibility testing (AST) of *Enterobacter cloacae* isolates.

Antibiotics	*Enterobacter cloacae* Strain 1		*Enterobacter cloacae* Strain 2		2022 EUCAST breakpoints (mg/L)
	AST	MIC (mg/L)	AST	MIC (mg/L)	
Piperacillin/tazobactam	R	-	R	-	
Cefepime	R	-	R	-	
Ertapenem	S	-	R	2^a^	0.5
Imipenem	S	-	S	0.25^a^	4
Meropenem	S	-	S	0.25^a^	8
Ceftazidime/Avibactam	-	-	S	0.5^a^	8
Meropenem/Vaborbactam	-	-	S	0.06^a^	8
Imipenem/Relebactam	-	-	S	0.06 ^b^	2
Tigecycline	-	-	R	1.5^a^	0.5
Fosfomycin	R	-	R	-	
Sulfamethoxazole-trimethoprim	R	-	R	-	
Ofloxacin	R	-	R	-	
Ciprofloxacin	R	-	R	-	
Amikacin	S	-	S	-	
Gentamicin	R	-	R	-	
Tobramycin	R	-	R	-	

MIC: minimum inhibitory concentration. S: Sensitive. R: Resistant. EUCAST: European Committee on Antimicrobial Susceptibility Testing. ^a^MICs determined with Etest^®^ (bioMérieux, Marcy-l’Etoile, France), ^b^MICs determined with MIC test strip (MTS) (Liofilchem, Inc., Waltham, MA).

**FIGURE 2:**
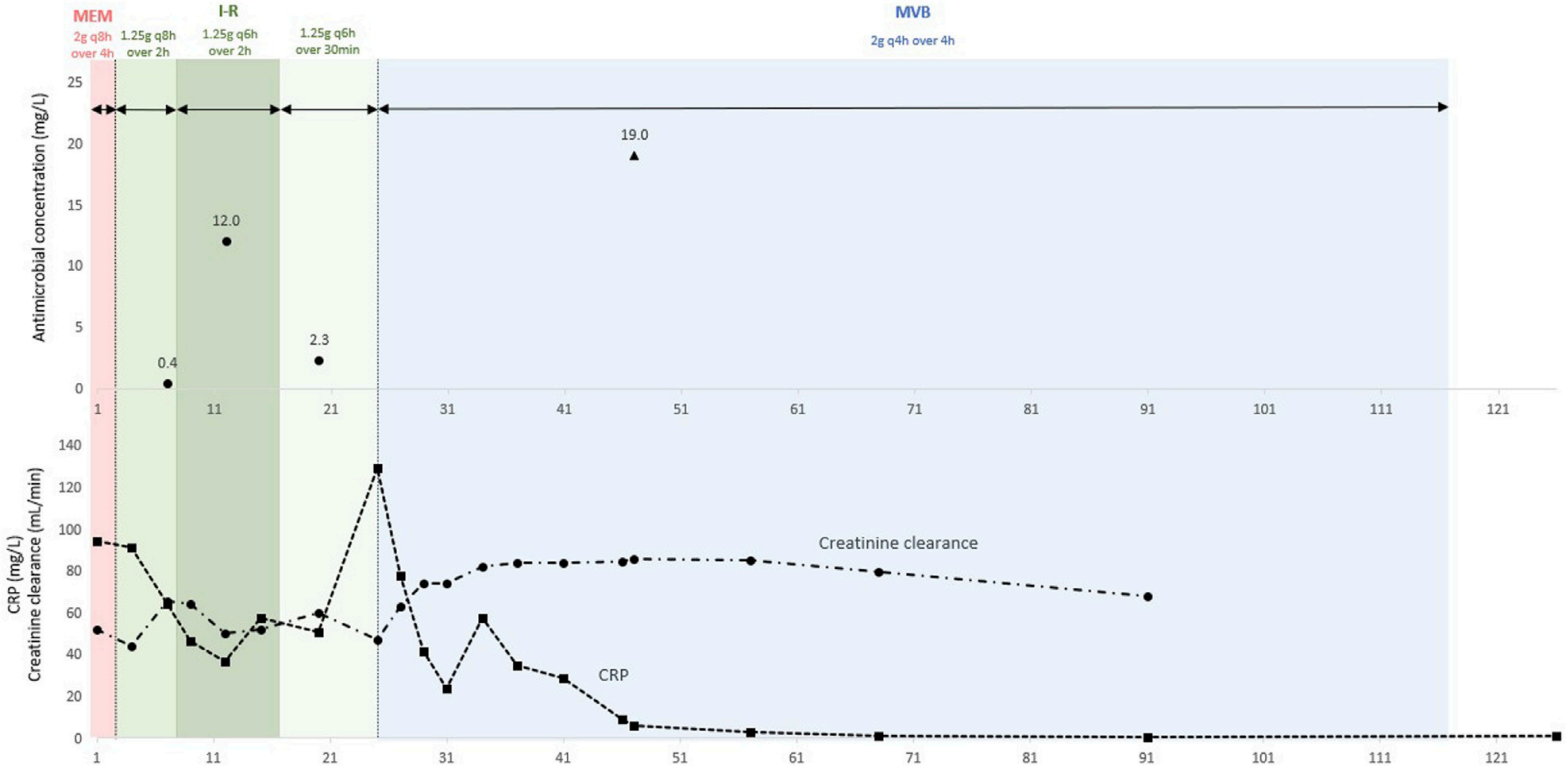
Therapeutic drug monitoring of imipenem and meropenem plasma concentrations in the patient. Imipenem through concentrations are represented by black circles and meropenem steady-state concentrations are represented by black triangles. Creatinine clearance is represented by black circles and dotted line, and C-reactive protein (CRP) is represented by black squares and dotted line.

Among the available therapeutic options tested, only the novel β-lactam-β-lactamase inhibitor agents, namely, ceftazidime/avibactam (CZA), I-R and MVB remained active against the carbapenem-resistant *E. cloacae* strain (Table 1). On the one hand, given the presumed toxicity of meropenem, we initially decided not to use MVB. On the other hand, I-R had the lowest minimum inhibitory concentration (MIC), at 0.06 mg/L, more than four times lower than the MIC of meropenem (0.25 mg/L) and eight time lower than the MIC of CZA (0.5 mg/L), see Table 1. A treatment with I-R was therefore administered at 1.25 g (*i.e.* 500 mg of imipenem, 500 mg of cilastatin and 250 mg of relebactam) as a 2 h infusion every 8 h. As the renal function improved (eGFR = 65 mL/min) we performed a therapeutic drug monitoring (TDM) of imipenem 7 days after I-R initiation. Blood samples for imipenem plasma concentration measurement were collected 15–30 min prior to the start of the subsequent infusion (trough or Cmin levels), then imipenem concentration were assessed utilizing an ultra-high performance liquid chromatography technique combined with high-resolution mass spectrometry as previously described (Bouglé et al., 2019). Plasma trough concentration were at 0.4 mg/L (Figure 2; Table 2). We then increased the I-R dose at 1.25 g q6h over 2 h, which allowed plasma through concentration of imipenem to reach 12 mg/L at day 12 (Figure 2; Table 2). The patient was afebrile, and the CRP level decreased to 37 mg/L. He was discharged to a rehabilitation centre.

**TABLE 2:** Pharmacokinetic models parameter estimates.

	Plasma concentration (mg/L)	Clearance (L/h)	Distribution volume (L)
I-R sample 1	0.4	9.39	12.6
I-R sample 2	12	8.19	12.6
I-R sample 3	2.3	9	12.6
MVB	19	13.04	12.02

PK parameters were estimated using R software version 4.2.0 (The R Foundation for Statistical Computing, Vienna, Austria) with PKPDsim version 1.3.0 and tidyverse version packages. I-R:imipenem/cilastatin/relebactam; MVB: meropenem/vaborbactam

During his stay in the rehabilitation centre, I-R dosing regimen was changed in 1.25 g as a 30 min infusion every 6 h and the TDM performed at day 20 days found an imipenem plasma through concentration of 2.3 mg/L (Figure 2; Table 2). At day 25, as the patient experienced a relapse in thoracic back pain, and CRP levels rose to 130 mg/L, he was readmitted to the infectious diseases department (Figure 2). A new spinal CT-scan showed severe osteolysis of T11-T12 with a significant posterior wall recession, resulting in a 60% central canal stenosis. At this time, we discussed two antimicrobial strategies: restarting the extended-infusion I-R over 2 h or modifying the treatment in favor of continuous infusion of MVB. As the patient renal function continued to improve (eGFR = 85 mL/min), we selected MVB 2 g (meropenem 1 g and vaborbactam 1 g) q4h as a 4-h infusion, resulting in a continuous infusion of 12 g/day of MVB. Surgical laminectomy of T11-T12 and percutaneous osteosynthesis of T9-T10 and L1-L2 were performed on day 36, to relieve central canal stenosis responsible for the pain the patient. No surgical debridement could be performed due to the spinal instability caused by the extensive osteolysis of T11-12 and no orthopaedic hardware was implanted in T11-T12 to limit the risk of bacterial biofilm formation on such implants, which would hinder antimicrobial therapy. Microbiological cultures of the biopsies of the affected bone and intervertebral disc were sterile. Pain gradually decreased after the surgery. At day 47, 20 days after MVB initiation, CRP levels were at 6 mg/L. At the time, plasma concentration of meropenem was measured at 19 mg/L (Figure 2; Table 2), in a blood sample collected at steady-state using high-performance liquid chromatography coupled with Ultraviolet Detection as previously described (Larcher et al., 2023). Importantly, no sign of clinical or biological toxicity was evidenced during I-R or MVB treatment course. At day 50, the patient was afebrile with normal CRP levels and was discharged to a rehabilitation centre. One month after an antibiotic course of 90 days, the clinical outcome was favourable, and the CRP decreased to 0.5 mg/L (Figure 2).

## 3 Discussion

To the best of our knowledge, this is the first case of successful treatment of a vertebral osteomyelitis caused by carbapenem-resistant *E. cloacae* with continuous infusion of MVB at 2 g q4h as a 4-h infusion in a patient with normal renal clearance. This dosing regimen achieved plasma steady-state concentrations of meropenem up to 19 mg/L. In addition, our data also suggest the feasibility of extended-infusion of I-R as 1.25 g q6h over 2 h to achieve plasma through concentration of imipenem up to 12 mg/L. There were no adverse effects associated to prolonged treatment with I-R and MVB.

The treatment strategies reported herein were based on limited evidence. Few cases reported successful treatment of BJIs with novel antibiotics such as CZA (Rempenault et al., 2021; Davido et al., 2023) and I-R (Rebold et al., 2021; Larcher et al., 2022). Data on bone diffusion of ceftazidime, meropenem, and imipenem encourage their use in BJI treatments (Landersdorfer et al., 2009; Thabit et al., 2019), but no data on beta-lactamase inhibitors diffusion in bones and joints are available. However, β-lactamase inhibitors appear to have the same pharmacokinetics as the beta-lactam with which they are associated (Nicolau et al., 2015; Wenzler et al., 2017; Rizk et al., 2018) suggesting novel β-lactam/β-lactamase inhibitors could be used in treatment of BJIs. Additionally, these new antimicrobials offer the advantage of being less nephrotoxic compared to treatment regimens based on colistin (Wunderink et al., 2018; Brown et al., 2020). Recent data indicate that while colistin monotherapy for treating BJIs caused by MDR bacteria is associated with high toxicity and mortality rates, these rates are even higher when colistin is combined with fosfomycin (Katip et al., 2024). This highlights the challenges of managing colistin and underscores the urgent need to explore more effective and less toxic alternatives, such as I-R and MVB.

As β-lactams and β-lactamase inhibitors displays mainly time-dependent killing, either extended or continuous infusion are recognized options for optimizing the time during which the antimicrobials concentration is maintained above the MIC of the treated bacteria (Abdul-Aziz et al., 2020). Optimized administrations of antimicrobial aim to improve microbiological and clinical outcome in the setting of severe diseases caused by difficult to treat bacteria (Roberts et al., 2016; Abdul-Aziz et al., 2020; Le Vavasseur and Zeller, 2022). However, few data have been published on MVB administration by continuous infusion (Larcher et al., 2023) and none on the use of I-R extended infusion. This case report therefore represents the first data available in the literature, especially in the setting of BJIs.

To improve the safety and the efficiency of off-label use of I-R and MVB in extended or prolonged infusion, we complied with the product stabilities recommended by the manufacturers (2 h for I-R and 6 h for MVB) (Summary of product characteristics: Vaborem, 2018; Summary of product characteristics: Recarbrio, 2020), and carried out therapeutic monitoring of plasma concentrations of imipenem and meropenem using a high performance liquid chromatography method (Larcher et al., 2023). We report optimized dosing regimens of I-R and MVB that achieved plasma concentrations above the MIC of most susceptible pathogens throughout the whole dosing interval (and more than 100%ƒT>4xMIC for many pathogens) (Abdul-Aziz et al., 2020). These dosing regimens enable sufficiently high plasma concentrations to be achieved, making it possible to treat infections at sites where antibiotic distribution is limited, such as BJIs (Landersdorfer et al., 2009; Thabit et al., 2019). It should be noted that surgical debridement plays an important part in achieving clinical cure, in addition to antimicrobials, especially in case of MDR bacteria, in order to reduce bacterial inoculum (Papadopoulos et al., 2019). However, in our case, surgical intervention was necessary due to spinal instability. The surgery was not specifically intended to control the source of infection but rather for mechanical reasons, as the infection was considered controlled prior to the intervention. It is also important to note that the surgical bone biopsy was negative in culture, which provides further evidence of the microbiological effectiveness of the treatments used in this case report.

Our work has limitations. First, this is a single case report, and larger-scale studies are needed to confirm our results. Second, TDM of I-R and MVB was based solely on the plasma concentration of beta-lactams (namely, imipenem and meropenem). However, as beta-lactamase inhibitors have the same pharmacokinetics as the beta-lactam with which they are associated (Nicolau et al., 2015; Wenzler et al., 2017; Rizk et al., 2018), the plasma concentration of the later can be used as a surrogate. Nonetheless, we did not obtain I-R and MVB concentrations in bone biopsies to confirm the diffusion of these new antibiotics in the affected bone, which would have been the preferred method of TDM. Last, TDM of beta-lactam is not performed every day in our hospital, and due to this fact, we were unable to confirm the meropenem overdose suspected at the beginning of the patient care.

Despite the limitations inherent to the level of evidence provided by case reports, we believe that documenting novel treatment strategies (Tamma et al., 2022) is urgently needed to address the growing challenge of antimicrobial resistance (Murray et al., 2022). This case report, in particular, illustrates how healthcare professionals, through interdisciplinary collaboration, can pave the way for novel therapeutic approaches and propose innovative solutions for managing the most challenging infections caused by MDR bacteria. Nonetheless, it remains necessary to validate these strategies on a larger scale through well-conducted clinical studies, ideally randomized controlled trials.

In conclusion, the case reported here suggest that I-R extended infusion and MVB continuous infusion are promising options for treatment of BJI caused by non-CP-CRE and CPE, provided that susceptibility to these agents is confirmed by antibiotic susceptibility testing. Further studies are essential to assess the pharmacokinetics of these β-lactam-β-lactamase inhibitor associations in BJIs. Notably, data on simultaneous measurements of β-lactam and β-lactamase inhibitor concentrations in both plasma and bone, using various administration strategies, particularly extended or continuous infusions, are needed. Additionally, clinical studies are required to validate the use of I-R and MVB for treating BJIs caused by MDR bacteria and to confirm that protocols derived from pharmacokinetic studies are effective and safe in such indications.

## Data Availability

The original contributions presented in the study are included in the article/supplementary material, further inquiries can be directed to the corresponding author.
